# Future Perspectives on the Role of Stem Cells and Extracellular Vesicles in Vascular Tissue Regeneration

**DOI:** 10.3389/fcvm.2018.00086

**Published:** 2018-07-03

**Authors:** Eoghan M. Cunnane, Justin S. Weinbaum, Fergal J. O'Brien, David A. Vorp

**Affiliations:** ^1^Department of Bioengineering, University of Pittsburgh, Pittsburgh, PA, United States; ^2^McGowan Institute for Regenerative Medicine, University of Pittsburgh, Pittsburgh, PA, United States; ^3^Tissue Engineering Research Group, Department of Anatomy, Royal College of Surgeons in Ireland, Dublin, Ireland; ^4^Department of Pathology, University of Pittsburgh, Pittsburgh, PA, United States; ^5^Trinity Centre for Bioengineering, Trinity College Dublin, Dublin, Ireland; ^6^Advanced Materials and Bioengineering Research Centre, Royal College of Surgeons in Ireland and Trinity College Dublin, Dublin, Ireland; ^7^Department of Surgery, University of Pittsburgh, Pittsburgh, PA, United States; ^8^Department of Cardiothoracic Surgery, University of Pittsburgh, Pittsburgh, PA, United States; ^9^Department of Chemical and Petroleum Engineering, University of Pittsburgh, Pittsburgh, PA, United States

**Keywords:** tissue engineered vascular grafts, stem cells, autologous, allogeneic, conditioned media, extracellular vesicles, exosomes

## Abstract

Vascular tissue engineering is an area of regenerative medicine that attempts to create functional replacement tissue for defective segments of the vascular network. One approach to vascular tissue engineering utilizes seeding of biodegradable tubular scaffolds with stem (and/or progenitor) cells wherein the seeded cells initiate scaffold remodeling and prevent thrombosis through paracrine signaling to endogenous cells. Stem cells have received an abundance of attention in recent literature regarding the mechanism of their paracrine therapeutic effect. However, very little of this mechanistic research has been performed under the aegis of vascular tissue engineering. Therefore, the scope of this review includes the current state of TEVGs generated using the incorporation of stem cells in biodegradable scaffolds and potential cell-free directions for TEVGs based on stem cell secreted products. The current generation of stem cell-seeded vascular scaffolds are based on the premise that cells should be obtained from an autologous source. However, the reduced regenerative capacity of stem cells from certain patient groups limits the therapeutic potential of an autologous approach. This limitation prompts the need to investigate allogeneic stem cells or stem cell secreted products as therapeutic bases for TEVGs. The role of stem cell derived products, particularly extracellular vesicles (EVs), in vascular tissue engineering is exciting due to their potential use as a cell-free therapeutic base. EVs offer many benefits as a therapeutic base for functionalizing vascular scaffolds such as cell specific targeting, physiological delivery of cargo to target cells, reduced immunogenicity, and stability under physiological conditions. However, a number of points must be addressed prior to the effective translation of TEVG technologies that incorporate stem cell derived EVs such as standardizing stem cell culture conditions, EV isolation, scaffold functionalization with EVs, and establishing the therapeutic benefit of this combination treatment.

## Introduction

**Figure 1:**
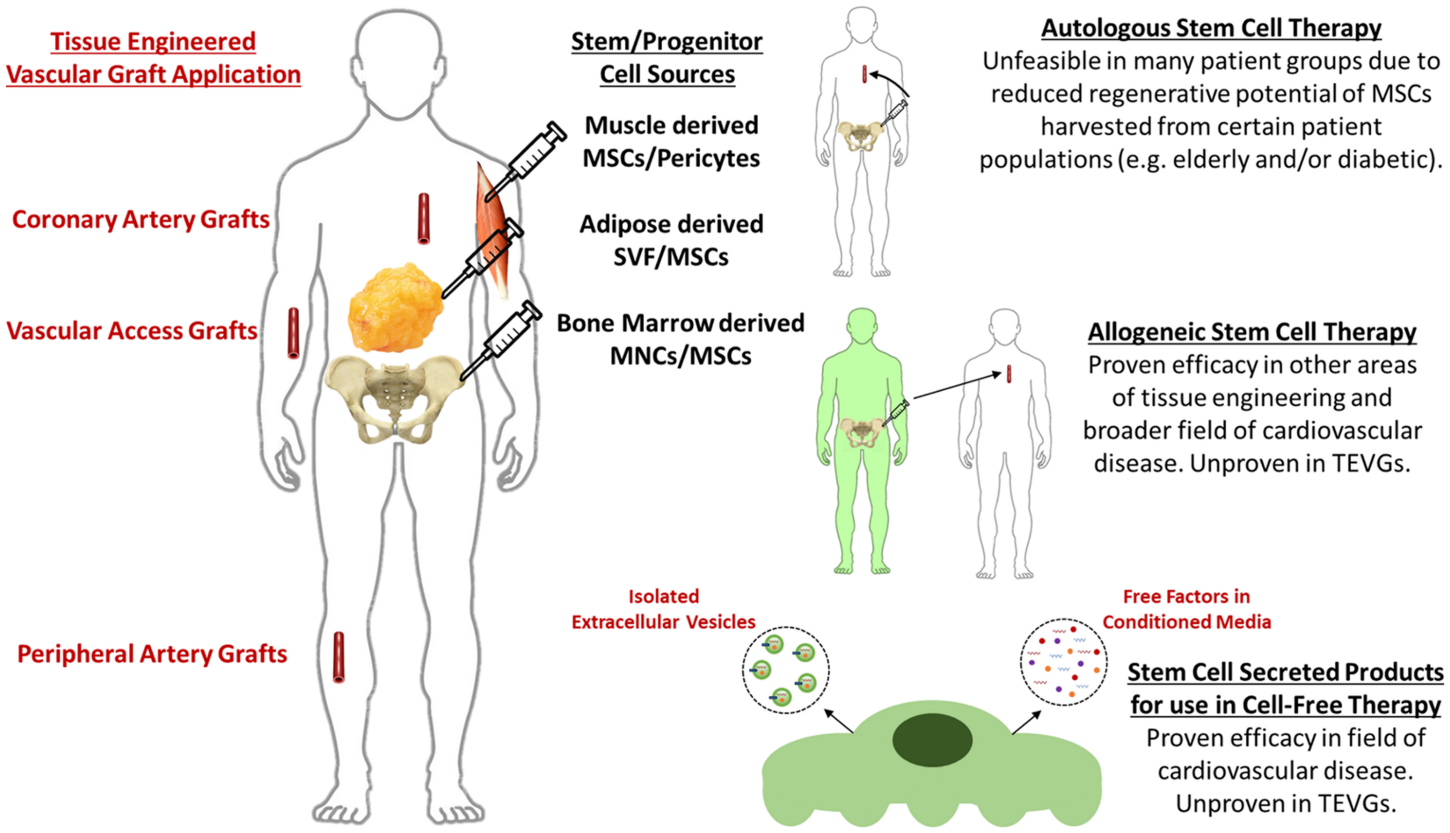
Current methods and future perspectives for stem cell-based tissue engineered vascular grafts.

Vascular tissue engineering is an area of regenerative medicine that attempts to restore defective segments of the vascular network. One approach to vascular tissue engineering is to implant biodegradable tubular scaffolds seeded with appropriate cells. Research has focused on lining the lumen of the scaffold with endothelial progenitor cells (1–5), self-assembly of vascular grafts by *in vitro* culture of fused vascular cell sheets (6–12), seeding scaffolds with native vascular cells (13–16), progenitor cells pre-differentiated into vascular phenotypes (17–22) using biomechanical/biochemical stimuli [as reviewed in Maul et al. (23)], and pluripotent stem cells pre-differentiated into vascular phenotypes (24, 25). However, employing native vascular cells, terminally differentiated progenitor/pluripotent cells, or self-assembled cell sheets requires extended culture periods and the use of expensive culture media that is often derived from xenogeneic sources. Seeding biodegradable scaffolds with undifferentiated stem (and/or progenitor) cells initiates scaffold remodeling through paracrine signaling to endogenous cells (26, 27). Seeding vascular scaffolds with stem cells also bypasses many of the aforementioned limitations due to the fact that a sufficient number of implant-ready cells can be acquired from a single harvest, therefore eliminating the time and resources spent culturing or differentiating cells. *The motivation for this review is that stem/progenitor cells have received an abundance of attention in recent literature regarding the mechanism of their paracrine therapeutic effect. However, this parallel research has yet to translate fully to the field of vascular tissue engineering. Therefore, the scope of this review includes the current state of TEVGs generated using the incorporation of stem cells in biodegradable scaffolds and potential cell-free directions for TEVGs based on stem cell secreted products* (Figure 1).

## Stem cell based TEVG studies

Numerous studies have demonstrated that implanting biodegradable vascular scaffolds, seeded with stem cells from a variety of sources, triggers the development of functional, immuno-compatible, native-like vascular replacements (Table 1). Bone marrow mononuclear cells (BM-MNCs) have been employed in numerous preclinical (26, 28–31, 33, 36–38, 43, 44) and clinical studies (28, 32, 51, 52). BM-MNCs are a heterogeneous population comprised of mesenchymal stem cells (MSCs), endothelial precursor cells, mature endothelial cells, hematopoietic stem cells, monocytes, CD4+ T cells, CD8+ T cells, B cells, and natural killer cells (26). Recently, it has been shown that BM-MNCs have a dose dependent effect on scaffold development when implanted as an inferior vena cava interposition in a mouse model whereby increasing BM-MNC number increased graft patency and decreased the number of infiltrated macrophages (42). Purified MSCs have also been employed in vascular tissue engineering and are obtained from various sources. MSCs are adherent adult progenitor cells with the ability to self-renew and differentiate into a variety of cells with a more specialized function [as reviewed in Huang and Li (53)]. Furthermore, MSCs secrete a variety of angiogenic and arteriogenic growth factors and cytokines (as discussed in section Allogeneic MSCs). Recent literature suggests that MSCs could be renamed Medicinal Signaling Cells to emphasize that MSCs do not differentiate at the site of injury (and are therefore not true stem cells), but rather signal to endogenous cells to regenerate and/or replace the injured/absent tissue (54). Bone marrow derived MSCs (BM-MSCs), purified from BM-MNCs, have demonstrated favorable preclinical findings in TEVGs (45–47). Similarly, adipose derived MSCs (ADMSCs) (48, 55) and muscle derived MSCs (49, 56) have been used in TEVG studies. Studies employing pericytes are also included in this review (50) as they have been shown to express MSC markers and display the capacity for tri-lineage differentiation [as reviewed in Crisan et al. (57)].

**Table 1:** Studies that have implanted scaffolds seeded with stem cells as vascular grafts.

**References**	**Type**	**Origin**	**Model**	**Source**	**Implant**	**Duration**	**Patency**
(28)	BM-MNC	Canine	Beagle dog	Auto	IVC	2 years	100%
(29)	BM-MNC	Canine	Beagle dog	Auto	IVC	4 weeks	100%
(30)	BM-MNC	Canine	Beagle dog	Auto	IVC	6 months	100%
(31)	BM-MNC	Ovine	Lamb	Auto	IVC	6 months	100%
(32)	BM-MNC	Human	Human	Auto	CPC	5.8 years	100%
(33)	BM-MNC	Human	Immunodeficient mouse	Xeno	IVC	6 months	100%
(26)	BM-MNC	Human	Immunodeficient mouse	Xeno	IVC	24 weeks	100%
(27)	BM-MNC	Murine	C57BL/6 mouse	Syng	IVC	2 weeks	68%
(34)	BM-MNC	Human	SCID/bg mouse	Xeno	IVC	10 weeks	100%
(35)	BM-MNC	Murine	C57BL/6 mouse	Syng	IVC	6 months	100%
(36)	BM-MNC	Murine	C57BL/6 mouse	Syng	IVC	4 weeks	100%
(37)	BM-MNC	Murine	C57BL/6 mouse	Syng	IVC	7 months	72% Survival
(38)	BM-MNC	Ovine	Lamb	Auto	IVC	6 months	100%
(39)	BM-MNC	Ovine	Lamb	Auto	IVC	6 months	100%
(40)	BM-MNC	Murine	C57BL/6 mouse	Syng	IVC	2 weeks	78% (Filter Group)
(41)	BM-MNC	Unclear	C57BL/6 mouse	Unclear	IVC	8 weeks	Unclear
(42)	BM-MNC	Murine	C57BL/6 mouse	Syng	IVC	2 weeks	95% (10 × 10^6^ cells Group)
(43)	BM-MNC	Murine	C57BL/6 mouse	Syng	IVC	2 weeks	88.9%
(44)	BM-MNC	Ovine	Lamb	Syng/Auto	CaVC	6 months	25%
(45)	BM-MSC	Canine	Beagle dog	Auto	AA	6 months	100%
(46)	BM-MSC	Human	Nude mouse	Xeno	CA	35 days	100%
(47)	BM-MSC	Human	Athymic rat	Xeno	CA	60 days	100%
(48)	ADMSC	Human	Lewis rat	Xeno	AA	8 weeks	100%
(48)	ADMSC	Human	Lewis rat	Xeno	AA	8 weeks	100%
(49)	MD-MSC	Rat	Lewis rat	Syng	AA	8 weeks	65%
(50)	Pericytes	Human	Lewis rat	Xeno	AA	8 weeks	100%

*BM-MNC, Bone Marrow Mononuclear Cells; Auto, Autologous; IVC, Inferior Vena Cava; CPC, Cavopulmonary connection; Xeno, Xeongeneic; Syng, Syngeneic; CaVC, Caudal Vena Cava; AA, Abdominal Aorta; CA, Carotid Artery*.

## Autologous stem cells

**Figure 2:**
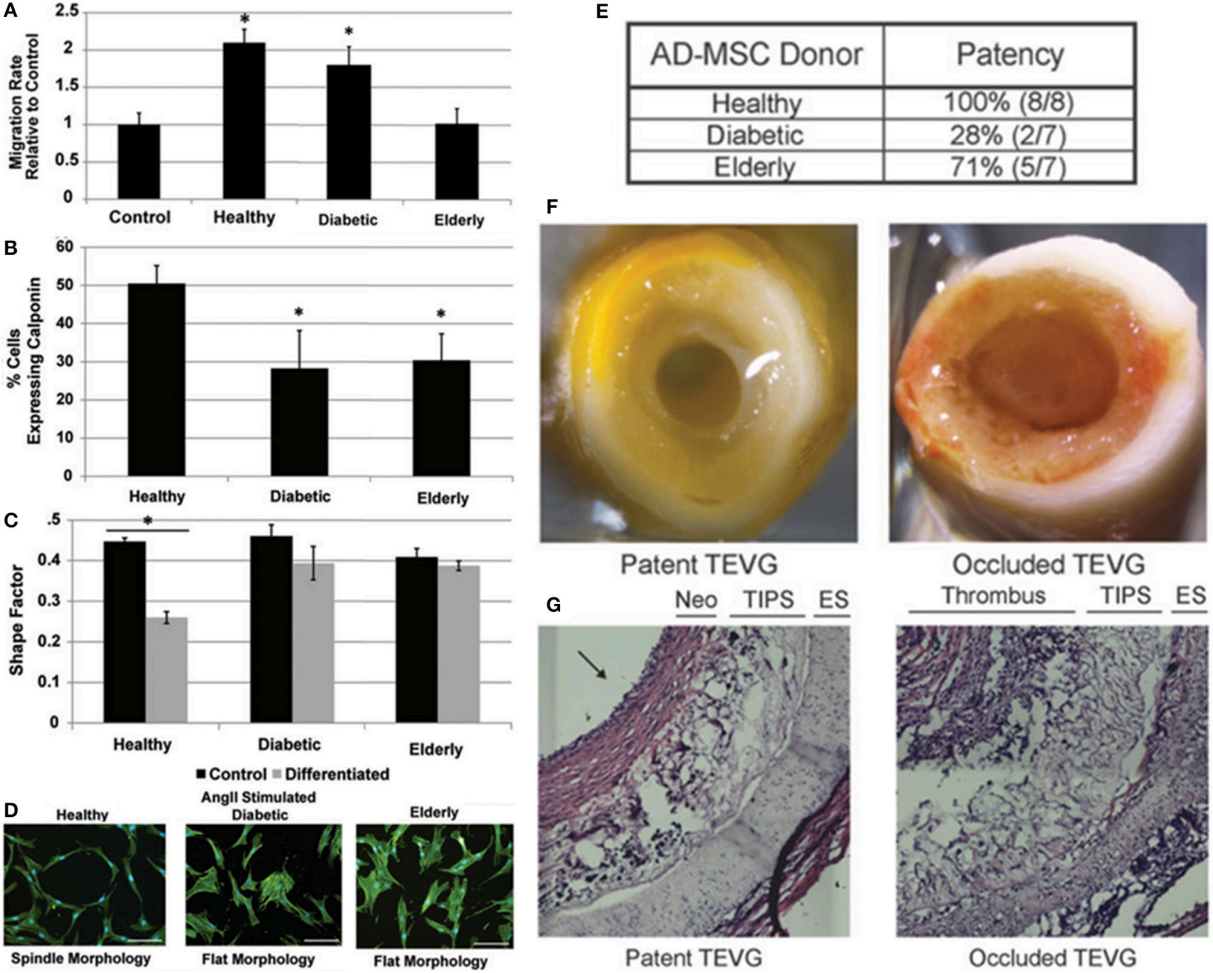
Combination of *in vitro* and *in vivo* studies that demonstrate the diminished regenerative potential of stem cells in vascular tissue engineering when harvested from elderly or diabetic patients. **(A–D)** The ability of ADMSCs from elderly or diabetic patients to encourage smooth muscle cell migration and secrete factors that promote fibrinolysis is decreased (48, 59). **(E–G)** The ability of ADMSCs to prevent acute thrombosis and encourage remodeling when seeded on a PEUU scaffold implanted in a murine model is reduced when harvested from elderly or diabetic patient groups (48). Adapted from Krawiec et al. (48, 59) with permission from Mary Ann Liebert, Inc.

Numerous preclinical (28–31, 38) and clinical studies (32, 51, 52, 58) have used autologous stem cells as a cellular base for vascular scaffolds. Autologous stem cell studies have focused on restoring vascular integrity in pediatric/young patients with congenital heart defects and have demonstrated favorable long term clinical results (32). However, a combination of *in vitro* and *in vivo* studies has demonstrated the diminished regenerative potential of stem cells in vascular tissue engineering when harvested from elderly or diabetic patients (Figure 2). The ability of ADMSCs to prevent acute thrombosis and encourage graft remodeling in a murine model is reduced when cells are harvested from elderly or diabetic patient groups and seeded on a PEUU scaffold (48) using established methods (60, 61). Furthermore, the ability of ADMSCs from elderly or diabetic patients to encourage smooth muscle cell migration and secrete factors that promote fibrinolysis is also decreased (48, 59). The work of Krawiec et al. therefore highlights the limitations of an autologous stem cell approach as many of the patient groups in need of regenerative therapies are elderly and/or diabetic e.g., coronary/peripheral bypass patients and end stage renal disease patients. Furthermore, the diminished regenerative potential of elderly/diabetic stem cells may expand to include more degenerative conditions. However, the inherent limitations of an autologous stem cell approach can be largely bypassed by using allogeneic MSCs harvested from young healthy donors.

## Allogeneic MSCs

MSCs are an appropriate candidate for allogeneic stem cell therapies as they are immune evasive. MSC immune evasion can be partially attributed to their low expression of major histocompatibility complex (MHC) class I antigens and freedom from expression of MHC class II antigens which are both associated with immune rejection (62, 63). Similarly, the MSC secretome has been shown to suppress immune response by inhibiting T cell proliferation and monocyte maturation and also by promoting regulatory T cells and M2 macrophages (64). Although allogeneic MSCs have not yet been investigated as a cellular base for TEVGs, the use of allogeneic fibroblasts for generating vascular grafts by self-assembly has been proven safe for use in humans as arteriovenous fistulas following devitalization (12). The use of allogeneic fibroblasts highlights the premise of employing allogeneic cells in vascular tissue engineering. Furthermore, other fields of tissue engineering have employed allogeneic MSCs to safely and effectively regenerate bone (65–67), cartilage (68–70), skin (71, 72), and nerve (73).

Clinical studies of allogeneic MSCs in the broader field of cardiovascular disease have also presented convincing evidence to suggest that allogeneic MSCs are minimally immunogenic and induce an equivalent therapeutic response when compared to autologous MSCs. The POSEIDON randomized trial compared the safety and efficacy of allogeneic MSCs to autologous MSCs in patients with ischemic cardiomyopathy. The trial results found that transendocardial delivery of allogeneic MSCs did not stimulate significant donor-specific immune reactions and was also associated with a reduction in left ventricle volume and an increase in ejection fraction comparable to treatment with autologous MSCs (74). It has also been demonstrated that transendocardial injections of allogeneic MSCs produce a dose dependent reduction in major adverse events in chronic heart failure patients (75). Furthermore, adventitial administration of commercially available allogeneic MSCs to the coronary arteries of myocardial infarction (MI) patients showed that allogeneic MSCs were well tolerated, with no serious adverse events, and significantly increased both ejection fraction and ventricular stroke volume (76). The safety and therapeutic efficacy of administering allogeneic MSCs to treat MI has also been demonstrated separately in both large (77) and small animal preclinical models (78). Combined, the preceding evidence supports the premise of employing allogeneic vascular cells as a cellular base for developing TEVGs and also the safety and efficacy of administering allogeneic MSCs to treat cardiovascular conditions. The use of allogeneic MSCs is therefore one potential future direction for stem cell based TEVGs that has yet to be fully investigated.

## Remodeling process of stem cell seeded vascular scaffolds

Despite the great success of directly incorporating stem cells in vascular tissue engineered scaffolds, evidence supporting a paracrine mechanism as the main effector of stem cell therapy indicates the potential of employing stem cell secreted products as a more straightforward, cell-free therapeutic base for tissue engineering (Figure 1). Compelling evidence for the paracrine effect of stem cells in vascular tissue engineering is that remodeling of implanted vascular scaffolds is mediated by an inflammatory process (26, 27, 42), and that seeded stem cells signal the recruitment and moderation of the immune cells that trigger the required inflammatory process in a paracrine manner (26, 27, 35).

The role of inflammation in vascular graft remodeling was initially demonstrated through observations that host monocyte and macrophage infiltration precedes the repopulation of scaffolds with vascular cells (26). Subsequently, it was demonstrated that peak macrophage infiltration coincides with the formation of functional vascular tissue and that depleting the host of macrophages completely inhibits the formation of neo-tissue (27). Furthermore, the role of modulating host immune cells was demonstrated by preventing host monocytes from secreting pro-inflammatory factors, through inhibition of TGF-β receptor 1, which significantly increased unseeded scaffold patency relative to untreated controls (79). Functionalization of vascular scaffolds to locally release TGF-β1 inhibitor was proven to be as effective as seeding BM-MNCs in promoting graft patency (41). Therefore, both recruitment and modulation of host immune cells are required to ensure the formation of a functional neo-vessel.

Evidence for the paracrine role of seeded stem cells in vascular scaffold remodeling is that seeded BM-MNCs reside in the scaffold for <7 days *in vivo* and are not incorporated into the developing neo-vessel (26, 35). Rather, the transient presence of BM-MNCs significantly increases the recruitment of host immune cells (monocytes and macrophages) compared to unseeded controls, partially through the secretion of MCP-1 (26). Subsequently, functionalization of vascular scaffolds to locally release MCP-1 was proven to be significantly more effective than seeded BM-MNCs in recruiting host monocytes (26). Furthermore, BM-MNCs have been shown to suppress the expression of M1 macrophage phenotype, the presence of which has been shown to decrease graft patency and remodeling (27). Seeded stem cells therefore modulate both the infiltration and phenotype of the host immune cells that mediate the vascular remodeling process in a paracrine manner through the secretion of bioactive products.

The concept of moving toward a cell-free approach by employing stem cell secreted products is to preserve the formation of neo-tissue while also removing the potential safety, regulatory, and practicality issues of cellular incorporation such as the use of stem cells with damaged/mutated DNA, undesirable trans-differentiation of persistent stem cells, and micro-vessel clotting in the case of stem cell relocation (80). It is important to note that TEVGs formed using cell secreted products may be better described as Tissue Regenerative Vascular Grafts to reflect the departure from the traditional cell based paradigm.

## MSC secreted factors/conditioned media

Stem cells, particularly MSCs, secrete a range of bioactive products that have an indirect or trophic effect on surrounding cells (81) and it has been proposed that these MSC-secreted bioactive products could replace MSCs as a therapeutic base for cell-free vascular tissue engineering (26, 41). One such manner of moving toward a cell-free approach is to utilize MSC conditioned media (MSC-CM) as it has been frequently demonstrated that MSCs secrete trophic factors into their surrounding media. However, the use of MSC-CM in vascular tissue engineering has demonstrated poor initial results. Scaffolds incubated over-night in BM-MNC-CM, following over-night incubation of the cells in serum-free media at 5% O_2_, exhibited poor patency rates *in vivo* which were comparable to PBS incubated scaffolds (patent: 2/10 vs. 6/25) (43).

Despite the discouraging results observed by Best et al, free injections of MSC-CM to treat MI have exhibited pre-clinical success. Intracardial administration of CM from BM-MSCs, under hypoxic conditions, and overexpressing the survival gene Akt1, significantly decreased infarct size and apoptosis in a murine model of MI (82). Intravenous and intracoronary administration of CM harvested from human embryonic stem cell (hESC) derived MSC was associated with a 60% reduction in infarct size, improvements in systolic and diastolic cardiac performance and increased capillary density in an infarct porcine model (83, 84). Furthermore, co-administration of MSC-CM and the parent ADMSCs synergistically increased neovascularization of infarcted myocardium compared to saline control in a porcine model of MI (77).

Culture conditions of MSCs can greatly alter the content of MSC-CM (85), therefore optimization of culture conditions could generate a more effective therapeutic. Exposing BM-MSCs to hypoxic conditions has been shown to induce a >1.5 fold increase in an array of angiogenic/arteriogeneic cytokine genes; furthermore, administering the same MSC-CM to a murine model of hind limb ischemia enhanced collateral flow, improved limb function, reduced auto-amputation, and attenuated muscle atrophy when compared with control media (86). Forming spheroids of BM-MSCs (25k cells) using the hanging drop method has been shown to increase the production of anti-inflammatory agents TSG-6 and PGE2 compared to dissociated MSCs, and the administration of the resulting MSC-CM attenuated macrophage phenotype *in vitro* and significantly lowered inflammation in a mouse model of peritonitis (87, 88). Exposing MSC spheroids to hypoxic conditions and inflammatory stimuli further enhanced the secretion of PGE2 and VEGF (85) and encapsulating MSC spheroids in unmodified or RGD modified fibrin gels has also been shown to increase MSC secretion of VEGF and PGE2 (89–91). However, recent findings suggest that MSCs seeded onto macroporous scaffolds secrete significantly higher levels of pro-angiogenic factors compared to MSCs encapsulated in fibrin gels (92). Furthermore, culturing ADMSCs on electrospun fibers produces significantly higher levels of anti-inflammatory and pro-angiogenic cytokines compared to those cultured on plates (93). Combined, the preceding evidence suggests that culturing MSC spheroids in 3D hypoxic environments, and exposing cells to an inflammatory stimulus, enhances the anti-inflammatory and pro-angiogenic potential of the resulting MSC-CM.

Providing a therapeutic effect using MSC secreted factors or MSC-CM is limited by the difficulties in delivering these products to the intended cell type and also by their short residence time *in vivo* which necessitates high initial concentrations. These limitations can largely be overcome by employing only the extracellular vesicles (EVs) secreted by stem cells. EVs offer many benefits as a therapeutic base for functionalizing vascular scaffolds such as cell specific targeting via the presentation of surface/membrane proteins (94, 95), physiological delivery of cargo to target cells (96, 97), reduced immunogenicity and stability under physiological conditions including protection of cargo from enzymatic degradation (98) and bypass of compliment activation (95, 99, 100). The potential of employing EVs as a therapeutic base for functionalizing vascular scaffolds is explored in the following section.

## MSC derived extracellular vesicles

**Figure 3:**
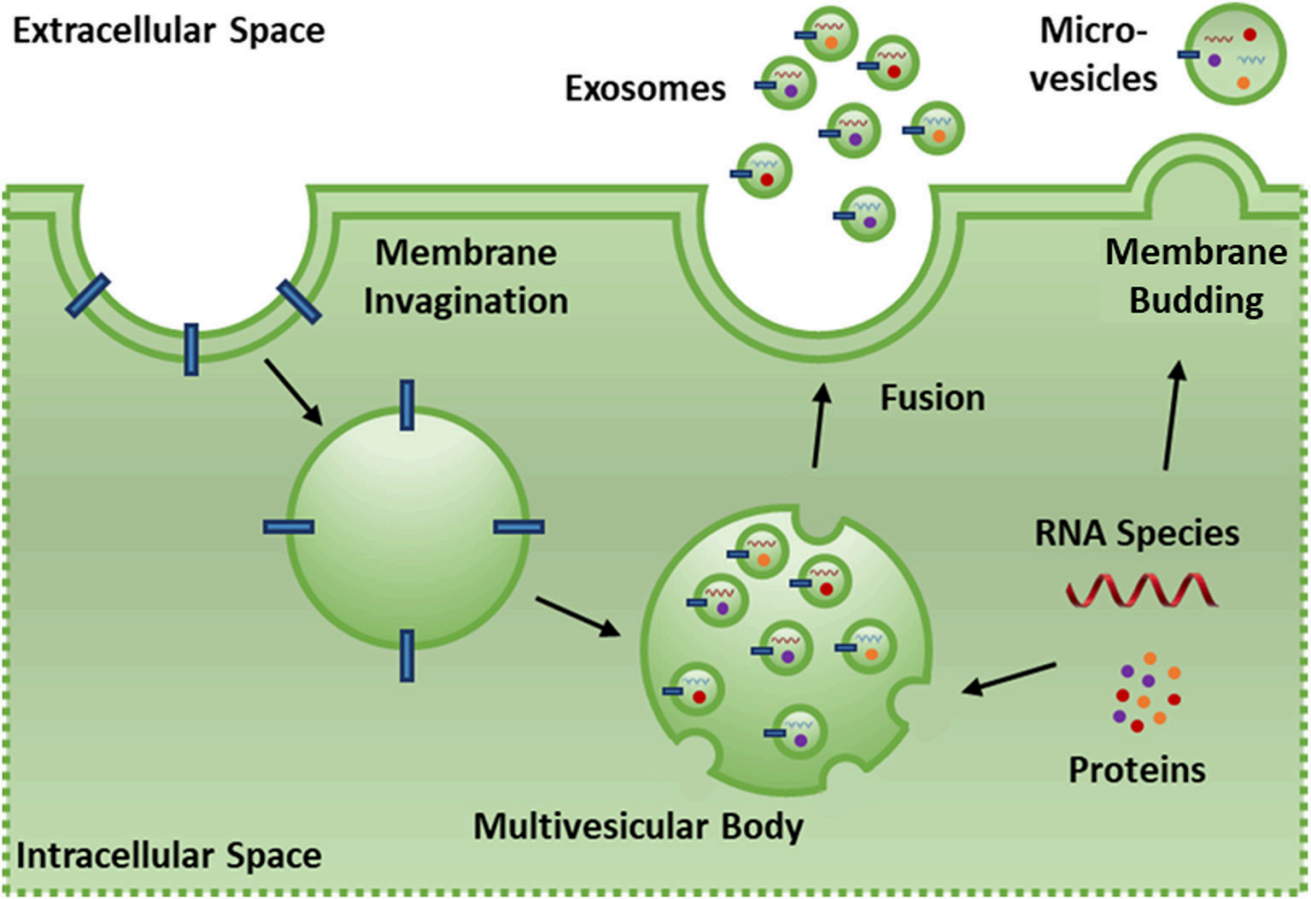
Exosomes (30–200 nm) are released by cells when intracellular multi-vesicle bodies form via invaginations of the cell membrane and are selectively loaded with endosomes containing protein, mRNA and miRNA. Fusion of the multi-vesicle body with the cell membrane releases these endosomes as exosomes. Micro-vesicles (200–1,000 nm) are released via direct outward budding of the cell membrane and contain protein, mRNA and miRNA. The loading of microvesicle cargo is less selective than exosomes and membrane proteins are more reflective of the parent cell membrane due to direct budding.

EVs are cell-derived phospholipid membrane based nano-particles that present with functional surface/membrane proteins and contain protein and RNA species that dynamically reflect the state of the parent cell and tissue (101). EVs are produced by most cells in the body (102, 103) and serve to transmit biological signals, transfer proteins/nucleic acids, and induce biological effects on target cells via surface receptor interactions, membrane fusion or endocytosis of the EVs by the target cell (96, 97). EVs can be categorized into three classes based on their cellular origins. Exosomes (30–200 nm) are released by cells when intracellular multi-vesicle bodies form via invagination of the cell membrane and are selectively loaded with endosomes containing protein, mRNA and miRNA. Fusion of the multi-vesicle body with the cell membrane releases these endosomes as exosomes (104). Micro-vesicles (200–1,000 nm) are released via direct outward budding of the cell membrane and contain protein, mRNA, and miRNA (Figure 3). The loading of microvesicle cargo is less selective than exosomes and membrane proteins are more reflective of the parent cell membrane due to direct budding (103). Apoptotic bodies (1,000–5,000 nm) are released by cells upon fragmentation of the plasma membrane during apoptosis (105). The term EV is used here to refer exclusively to exosomes and microvesicles as apoptotic bodies are distinct in activity and content (106).

EVs can be isolated from cell culture supernatant via density-based, size-based, precipitation, immunoaffinity, and microfluidic based techniques [as reviewed in Li et al. (107)]. Although ultracentrifugation remains the gold standard, each technique has inherent advantages and limitations regarding process speed/cost and EV yield/functionality. Once isolated, guidelines have been published by the International Society for EVs regarding minimum standards for EV characterization. These guidelines require that EV size, concentration and morphology be determined in addition to screening for EV enriched markers and quantifying the co-precipitating protein levels to assess the purity of the EV isolate (108).

MSC-EVs have already shown regenerative potential and have also been credited with many of the therapeutic effects seen during the treatment of cells and tissues with MSC-CM (109, 110). Furthermore, MSCs are regarded as the optimal source for obtaining therapeutic EVs due to their immunomodulatory properties (111), their high expansion capacity/potential for immortalization (112) and the large numbers of EVs that they secrete relative to other cells (100). Although not yet employed in the field of vascular tissue engineering, other areas of tissue engineering have begun to utilize EVs such as bone regeneration (113), adipose tissue regeneration (114), and wound healing (115, 116). Furthermore, the functional relevance of EVs in regenerative medicine (such as promoting cell viability, angiogenesis, extracellular matrix interactions, and immunomodulation) has already been highlighted [as reviewed in De Jong et al. (117)].

**Figure 4:**
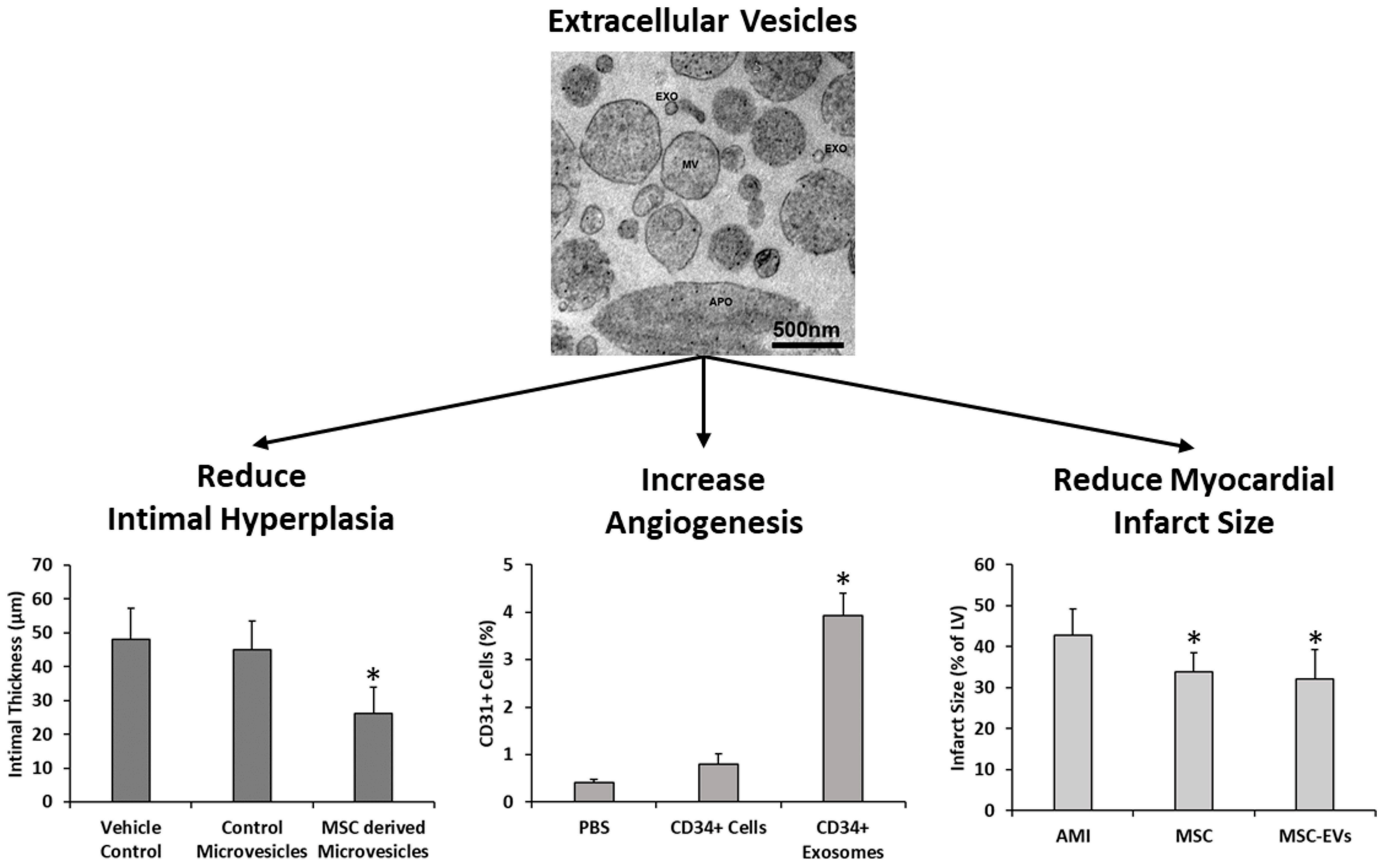
MSC derived extracellular vesicles have been previously shown to reduce intimal hyperplasia in murine models of vein grafting relative to vehicle and fibroblast microvesicle controls (118), increase angiogenesis in murine matriplug models relative to vehicle control and parent cells (119), and also to reduce infarct size in murine models of MI in a manner similar to the parent cells (124). EXO, exosomes; MV, microvesicles and APO, apoptotic bodies. Images and data adapted from Liu et al. (118), Sahoo et al. (119), Bian et al. (127), and Osteikoetxea et al. (131) with permissions. ^*^denotes statistically significant differences at *p* < 0.05.

Numerous studies have examined the effects of free EV injections in other areas of cardiovascular research such as vein grafting, angiogenesis and MI. Liu et al. demonstrated that the degree of intimal hyperplasia was significantly decreased following vein graft implantation in a murine model with multiple intraperitoneal injections of human ADMSC-EVs. Macrophage presence was also found to be reduced and significantly decreased expression levels of IL-6 and MCP-1 were found in ADMSC-EV treated mice compared to controls (118). MSC derived exosomes have been shown to induce angiogenesis *in vitro* through increased endothelial cell migration and tube formation (119, 120), and also *in vivo* through increased vessel formation in murine Matrigel plugs, corneal assays, and cerebral artery occlusion relative to controls (119, 121–124). Furthermore, administration of hESC derived exosomes (125), hESC-MSCs derived exosomes (109, 112, 126), BM-MSC derived EVs (127), and BM-MSC derived exosomes (128–130) have all been shown to significantly reduce infarct size in murine models of MI compared to controls (Figure 4). Interestingly, only intact and not lysed exosomes demonstrated a therapeutic effect, therefore suggesting that both exosome mediated delivery, in addition to exosome cargo, are required to successfully treat cardiovascular conditions (126).

In an attempt to elucidate the therapeutic mechanism of MSC-EVs, extensive transcriptomic and proteomic characterization of ADMSC-EVs has been performed and the results compared to those obtained from the parent MSCs. It has been shown that ADMSC-EVs contain a similar yet distinct protein, miRNA and mRNA cargo compared to their parent cells. Specifically, ADMSC-EVs are enriched for the mi-RNAs miR-183, miR-378, miR-140, and miR-222; for 255 genes including TRPS1, ELK4, KLF7, and NRIP1; and for 277 proteins that play important biological roles including glycoproteins, extracellular matrix remodeling, blood coagulation, inflammatory response, TGF-B signaling pathway, and angiogenic proteins. The ADMSC-EV cargo is therefore enriched to support a range of functions important to vascular tissue engineering including extracellular matrix remodeling, angiogenesis, inflammation, blood coagulation, and apoptosis (132–134). Consequently, MSC-derived EVs are worthy of future research/therapeutic focus in this context.

## Future of MSC-EVs in vascular tissue engineering

The role of MSC-EVs in vascular tissue engineering is particularly exciting due to the need for a cell-free therapeutic base that can be incorporated into a scaffold and signal to cells in a paracrine manner to prevent acute thrombosis and encourage appropriate remodeling. However, a number of points must be addressed prior to the effective translation of TEVG technologies that incorporate MSC-EVs:

The optimal culture conditions for parent MSCs must be identified to ensure that the optimal yield of EVs is being obtained in a safe and repeatable manner. Although this has been studied extensively for MSC-CM (see section MSC Derived Extracellular Vesicles), the therapeutic effects of free factors and EVs in MSC-CM must be de-coupled and culture conditions optimized to specifically increase the therapeutic efficacy of isolated EVs.

A cheap, reliable and EV friendly method of isolating MSC-EVs must be identified and implemented to ensure that the optimal yield of EVs is being obtained in a safe and repeatable manner. Ultimately, a preferred method of isolating intact EVs must be identified and scaled so that EV based TEVGs can be developed into a clinically viable therapy.

The optimal method of delivering and retaining MSC-EVs into a tissue engineered vascular scaffold must be identified to ensure that MSC-EVs are present in sufficient numbers and remain intact. Encouraging research has demonstrated that directly incorporating EVs into a decalcified bone matrix scaffold is possible and elicits an equivalent neo-vessel formation response compared to incorporating MSCs alone following subcutaneous murine implants (113). Furthermore, it has been shown that cardiosphere derived EVs remain stable at −80°C for up to 90 days and that both *in vitro* and *in vivo* bioactivity is preserved following lyophilisation (135). This suggests that EVs can be directly incorporated into many forms of scaffold production and therefore exhibit potential as a therapeutic source in off-the-shelf vascular graft applications.

The *in vivo* remodeling potential of MSC-EV seeded vascular scaffolds must be assessed using established small and large animal preclinical models to determine if they elicit an appropriate TEVG host remodeling response.

## Summary

One approach to vascular tissue engineering is to implant biodegradable tubular scaffolds, seeded with autologous stem cells that trigger the development of functional native-like vascular replacements. However, stem cells harvested from elderly or diabetic patients have diminished regenerative potential in vascular tissue engineering. The inherent limitations of an autologous stem cell approach can be addressed using allogeneic MSCs. However, potential safety, regulatory, and practicality issues of cellular incorporation suggest that a cell-free approach may be more prudent. MSC-EVs present as one such cell-free approach and offer many benefits as a therapeutic base for functionalizing vascular scaffolds such as cell specific targeting, physiological delivery of cargo to target cells, reduced immunogenicity, and stability under physiological conditions. Despite promising findings of EV therapy in the broader field of cardiovascular research, further work is required to explore the full potential of this promising therapeutic in vascular tissue engineering.

## Author contributions

EC: concept generation, literature review, manuscript writing, critical review of manuscript; JW, FO and DV: concept generation, manuscript writing, critical review of manuscript.

### Conflict of interest statement

The authors declare that the research was conducted in the absence of any commercial or financial relationships that could be construed as a potential conflict of interest.
